# Fundamental Movement Skills and Physical Activity of 3–4-Year-Old Children within Early Childhood Centers in New Zealand

**DOI:** 10.3390/children8090742

**Published:** 2021-08-27

**Authors:** Ajmol Ali, Claire McLachlan, Tara McLaughlin, Owen Mugridge, Cathryn Conlon, Karen Mumme, Tayla Knightbridge-Eager

**Affiliations:** 1 School of Sport, Exercise and Nutrition, Massey University, Auckland 0745, New Zealand; O.Mugridge@massey.ac.nz (O.M.); C.Conlon@massey.ac.nz (C.C.); K.Mumme@massey.ac.nz (K.M.); t.eager@massey.ac.nz (T.K.-E.); 2 Department of Sport Science and Physical Education, The Chinese University of Hong Kong, Shatin, N.T., Hong Kong, China; 3 Faculty of Education, University of Waikato, Gate 5 Hillcrest Road, Hamilton 3216, New Zealand; c.mclachlan@federation.edu.au; 4 School of Education, Federation University Australia, Ballarat, VIC 3353, Australia; 5 Institute of Education, College of Humanities & Social Sciences, Massey University Manawatū, Private Bag 11 222, Palmerston North 4442, New Zealand; T.W.McLaughlin@massey.ac.nz; 6 Kimi Hauora Wairau, Marlborough Primary Health Organisation (KHW MPHO), Blenheim 7201, New Zealand

**Keywords:** accelerometry, preschool, motor skills, Test of Gross Motor Development, TGMD-2, physical education, wellbeing

## Abstract

We sought to describe and explore relationships between fundamental movement skills (FMS) and level of physical activity (PA; light-, medium-, vigorous, and kCal/hour) in preschool children, aged 3–4-years-old, across four early childhood education (ECE) settings. Children’s FMS were assessed using the Test for Gross Motor Development-2 (TGMD-2; *n* = 81) and PA via accelerometers (*S* = 53). Eighty-four children participated, with 50 in both assessments. The TGMD-2 showed as the children got older, their locomotor skills (*p* < 0.001, r = 0.512) and object control motor skills (*p* < 0.001, r = 0.383) improved. Accelerometry showed children were primarily inactive at ECE (78.3% of the time). There were significant correlations between kCal/hour and light (*p* < 0.001, r = −0.688), moderate (*p* < 0.001, r = 0.599) and vigorous (*p* < 0.001, r_s_ = 0.707) activity, and between gross motor quotient and locomotor (*p* < 0.001, r = 0.798) and object control (*p* < 0.001, r = 0.367) skills. No correlation was observed between gross motor quotient and kCal/hour. To conclude, children in this cohort were primarily inactive during ECE center hours. Moreover, gross motor quotient was significantly correlated to locomotor and object control skills.

## 1. Introduction

Four-to-ten percent of children aged under 5-years-old in The Netherlands [1] and Australia [2,3] are not meeting physical activity (PA) guidelines (totaling 180 min of combined light-, moderate-, and vigorous-PA per day). Furthermore, 2-year-olds in New Zealand (NZ) spend 1.5 h/day in sedentary screen use [4], consequently reducing active time. Despite many countries, including NZ [5], having (nutrition and) PA guidelines, low levels of, and low intensity of, PA have been observed in childcare settings, indicating that children may not be fulfilling their PA requirements [1,2,6]. As such, evidence suggests that children are not performing sufficient PA to ensure improved bone density, aerobic fitness and motor skills [7,8,9]; thus, the assumption that toddlers and young children are naturally active [1,2] may be damaging to children’s wellbeing. Consequently, there is a need for specific PA guidelines that stipulate both intensity and duration of PA required to provide benefits to children’s health, especially where there are currently no or non-specific guidelines for preschoolers [10].

Compared to children at home, children in childcare are less likely to engage in PA [11,12,13]. As most NZ children aged 3- and 4-years-old (86% and 92%, respectively) are enrolled in early learning services [14], these environments are important for the promotion of practices that contribute to children’s wellbeing [6,15]. United Nations International Children’s Emergency Fund (UNICEF) Innocenti [16] ranked NZ 35th out of 38 OECD and EU countries for overall child wellbeing (38th for mental well-being, 33rd for physical health, and 22nd for skills), thus further highlighting the critical need for NZ to have a stronger focus on childhood wellbeing.

As in other developed nations, overweight and obesity has become the norm in NZ [17]. Compared to children with a lower body mass index (BMI), children with a higher BMI are more sedentary, less physically active and less motor skill proficient [18]. The contributing factors to overweight/obesity are complex but not separate to NZ’s obesogenic environment where social factors encourage physical inactivity and the consumption of energy-dense, nutrient-poor foods [19]. These factors are identified as key drivers of excessive weight gain [20]; not coincidently, appropriate nutrition and PA are key health promotion strategies for reducing the risk of children becoming overweight [21].

Nearly one-third (30%) of NZ children, aged 2–14-years-old, are overweight or obese [22]. This is a non-significant reduction from 33% in 2014/15, and while this reduction is positive, it is too early to report a trend [22]. There continues to be health disparities among NZ children, with Pacific and Māori children, and children living in the most socioeconomically deprived areas, being 4.7, 1.6 and 2.7 times more likely to be obese as compared to non-Pacific and non-Māori children, and children living in the least deprived areas, respectively [22]. Therefore, determining culturally and contextually appropriate and effective interventions for preschool children, and especially Pacific and Māori children and children from socioeconomically deprived areas, is essential.

Evidence suggests that preschoolers’ physical opportunities are limited by rigid playground regulations [23,24]; insufficient space [25] and equipment [15,25]; teachers’ perceptions of risk [26]; beliefs about the opportunities that should be offered [23,27]; confidence in providing a range of PA opportunities, and limited knowledge, skills, professional learning and development (PLD) [27,28,29]. While teachers should be responsible for providing opportunities for PA and nutrition in the curriculum, there is a concerning gap in early childhood education (ECE) teacher and teacher education programmes, whereby nil to few hours may be allocated to cover PA [30,31]. Nevertheless, it is possible to increase teachers’ knowledge, skills, attitudes and intentional teaching, to promote PA and motor skills in ECE centers [15].

Increasing the likelihood of an active lifestyle has several benefits; PA and motor skill proficiency is associated with better physical, social and psychological development in childhood and adolescence [32,33]. Motor skill proficiency in children has been positively associated with time in moderate and moderate-to-vigorous intensity PA, and inversely associated with sedentary activity time [18,34]. Furthermore, as adolescents, children with better motor skill proficiency were 10–20% more likely to take part in vigorous PA [35], and have greater cardiorespiratory fitness [34], perceived sports competence [36,37] and cognitive skills [36].

Integrating health behavior into the curriculum has potential for increasing PA levels of preschoolers [38], and PA interventions have been seen to significantly improve fitness [39] and motor skills [39,40]. Appropriate motor skill interventions were effective in developing fundamental movement skill (FMS) in preschoolers, including those with developmental delays [41] and FMS delays in socioeconomically disadvantaged early education settings [42]. Altunsöz [41] recommends a minimum of 540 min of instructional time developing FMS and early intervention with developmentally appropriate programmes. Such interventions may increase future participation in PA as an outcome of perceived motor skill competence [41]. Thus, promoting PA and motor skill proficiency in young children could improve PA levels and intensity as children age.

Therefore, this study looks at the relationship between FMS and PA levels of preschool children, aged 3–4-years-old, attending Early Education Centers in New Zealand, to identify a potential avenue (FMS) to apply targeted interventions to improve PA levels of young New Zealanders.

The data reported in this paper are part of a larger study entitled “Physical Education in Early Childhood” (PEECh), the aim of which is to promote physical education and appropriate nutrition in ECE settings as a strategy to reduce obesity and increase children’s resilience. The aim of this study was to describe and explore relationships between motor skill proficiency and physical activity in preschool children, aged 3–4-years-old, within a sample of low socioeconomic status (SES) ECE centers in NZ.

## 2. Materials and Methods

### 2.1. Participants

This study includes data on 84 preschool children (56% male) with a mean ± standard deviation age of 4.02 ± 0.57 years old (Table 1). Eighty-one participants completed the Test of Gross Motor Development, 2nd Edition (TGMD-2; 56% male), and 53 completed accelerometry (62% male). The university ethics committee approved all procedures. The parents/caregivers (of the children who expressed an interest in the experiments) were fully informed about the aims, procedures and demands, and potential risks and discomforts of the study, before obtaining written consent. Furthermore, parents/caregivers were reminded of their right to withdraw their child from the study at any stage or time.

### 2.2. Study Design

#### 2.2.1. Fundamental Movement Skill Assessment

The TGMD-2 [43] was used to assess FMS. Suitable for 3–10-year-olds, the TGMD- uses skill-specific performance criteria to assess locomotor skills (e.g., running and hopping) and object control skills (e.g., kicking and throwing) [43].

Instructors demonstrated the TGMD-2 tasks (using specific criteria) on two occasions prior to the child making two attempts at each task. These data were collected by four instructors, each of whom underwent training moderated by a primary assessor using physical demonstration and verbal instruction, and with use of the TGMD-2 examiner’s manual and visual diagrams.

Locomotor and object control raw scores were tallied by the instructors and the TGMD-2 conversion tables [43] (pp. 54–60) were used to calculate the Standard Score, Percentile and Age Equivalent scores. Scoring was checked by the primary assessor, who was also responsible for scoring each child’s TGMD-2 assessment on all testing days.

The term ‘standard scores’ is used to describe raw data that have been converted, based on the specific age of the child; these scores allow comparison between locomotor and object control subtests. The percentage of the distribution equal to or below a particular score is as indicated by the percentile ranks (percentiles), for example, a percentile score of 75 means that 75% of the standardized sample scored at or below the examinee’s score. It is notable that these values are based on standardized percentile data from the US. ‘Developmental ages’ are age equivalents for tests of developmental abilities [43] and the TGMD-2 age equivalent data provide an estimation of how the subtest scores relates to typical age.

Results from a recent systematic review show that the TGMD-2 has “moderate-to-excellent internal consistency, good-to- test–retest excellent inter-rater reliability, good-to-excellent intra-rater reliability, and moderate-to-excellent reliability” [44]. Prior to visiting the centers, pilot testing was carried out to ensure the primary assessor recognized the successful completion or failure of each skill criterion. An additional instructor also scored the child’s TGMD-2 assessment periodically throughout the testing period to check the reliability and reproducibility of the primary assessor’s scoring.

Health and safety assessments were completed in each testing environment within the four centers to establish any health and safety risks before each TGMD-2 session took place. Rooms were selected based on the requirements of the locomotor and object control skills whilst also balancing noise levels, and interruptions and distractions that could occur during a child’s assessment.

#### 2.2.2. Physical Activity (Accelerometry)

Accelerometers were used to objectively measure PA and accurately measure sitting, standing, and movement time. The accelerometer (ActiLife 6.13.2; model wGT3X-BT, Actigraph, Pensacola, FL, USA) was attached to a belt placed around the child’s waist. The unit itself was attached over the child’s right hip. One of the researchers (O.M.) showed the early childhood educators how to correctly place the units on a child. ECE teachers were asked to place the units on a selected number of participants (due to available numbers of accelerometers) every weekday for a one-week period. Participants wore the accelerometers for the duration of time they were present at the ECE center, and teachers were tasked with removing the units before the child left the center. Data were analyzed using appropriate software (ActiLife 6.13.2; model wGT3X-BT Actigraph, Pensacola, FL, USA) and based on previously established activity models for preschool children [45,46].

#### 2.2.3. Statistical Analyses

Statistical analysis was performed using IBM^®^ SPSS^®^ software for Windows, version 25.0 (IBM SPSS Software, 2017; Chicago, IL, USA). Participant data are described using mean and standard deviation for normally distributed data, or median (25, 75 percentile) for non-normally distributed data. Categorical variables are summarized as frequencies and percentages. The Shapiro–Wilk test and normality plot were used to evaluate the normality of distributions. Student’s *t*-test, Mann–Whitney, ANOVA and Kruskal–Wallis explored relationships between TGMD-2 (locomotor skills, object control skills and gross motor quotient), accelerometry (levels of activity; light, medium and vigorous PA, and kcal/hour) and other variables (age and sex). Interactions between age and sex were tested using ANOVA. The post hoc Tukey test was used to explore multiple comparisons. Pearson’s correlation was used to examine relationships between two normally distributed quantitative variables and Spearman’s correlation coefficient was used where one or both variables were non-parametric. Statistical significance was set at *p* < 0.05 and accepted effect size metrics and interpretations were followed.

## 3. Results

A total of 84 children participated in the study, with 81 children involved in TGMD-2 assessment and 53 children undertaking accelerometry measurements (Table 1). Fifty children participated in both TGMD-2 and accelerometry assessments.

Compared to female preschool children, males showed significantly greater TGMD-2 object control skill scores (F = 5.116, (1, 79) df, *p* = 0.026, r *=* 0.25 (medium effect); Table 2). There were no other significant differences in TGMD-2 (Table 2) and accelerometry (Table 3) scores between male and female preschool children.

As the children got older, their locomotor skills (*p* < 0.001, r = 0.512; large effect) and object control motor skills (*p* < 0.001, r = 0.383; medium effect) improved. Age was significantly correlated to both the locomotor age equivalent (*p* < 0.001, r_s_ = 0.451; medium-large effect) and the object control age equivalent (*p* = 0.001, r_s_ = 0.372; medium effect). There were no other differences in TGMD-2 data and age.

**Figure 1:**
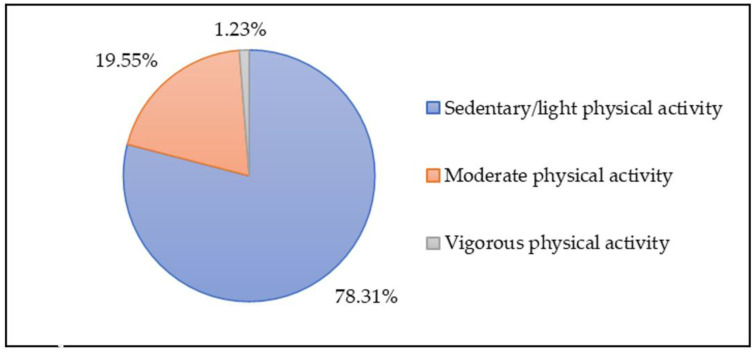
Percentage of recorded time engaged in different levels of activity, from accelerometers.

Activity levels, as measured by the accelerometers, are shown in Figure 1. The children were primarily inactive 78.31% of the time. Moderate and vigorous activity was 19.55% and 1.23% of the time measured. There were no differences in age or sex.

Whilst wearing accelerometers, time spent engaged in moderate-to-vigorous physical activity ranged from 0.19% to 41.75% of time recorded, with over half of preschoolers engaged in moderate-to-vigorous physical activity for at least 20% of the time (*n* = 30, 57%).

There were significant correlations between kCal/hour and light (*p* < 0.001, r = −0.688; large effect), moderate (*p* < 0.001, r = 0.599; large effect) and vigorous (*p* < 0.001, r_s_ = 0.707; large effect) activity. There were significant correlations between gross motor quotient and locomotor (*p* < 0.001, r = 0.798; large effect) and object control (*p* < 0.001, r = 0.3686; medium effect) skills. Therefore, it was decided to use kCal/hour and gross motor quotient as a proxy, for activity and skills, to explore whether skills can predict activity levels. No correlation was observed between gross motor quotient and kCal/hour (*n* = 50, *p* = 0.155, r = −0.204; small-medium effect).

There was no correlation between raw scores (TGMD-2 locomotor and TGMD-2 object control) and activity (sedentary/light, moderate, vigorous). There was, however, a correlation between locomotor standard scores and sedentary activity (*p* = 0.014, r = 0.344; medium effect), and locomotor standard scores and moderate physical activity (*p* = 0.031, r = −0.306; medium effect). Gross motor quotient was correlated with vigorous physical activity (*p* = 0.034; r_s_ = −0.301; medium effect).

## 4. Discussion

This study aimed to explore the relationship between motor skill and physical activity levels in 3–4-year-old preschool children in NZ. Our cohort was primarily inactive during ECE center hours (78.6% of time). Moreover, gross motor quotient was significantly correlated to locomotor and object control skills. As children got older, locomotor and object control skills significantly improved.

Overall, our students have greater [47], lower [48] or similar [49] gross motor quotient scores than international data, indicating that the preschoolers in this study are more, less or similarly proficient in overall FMS, respectively; our students had similar [49,50,51], greater [47] or lower [48] locomotor standard scores; and similar [48,49], greater [47] or lower [51] object control skills standard scores compared to previous international studies (Table 4).

Children in this study remained primarily inactive, spending only a subset, 21.36% (5.19, 27.43%), of time at the ECE, in moderate and vigorous PA. A study in Canada [52] also reported that during childcare, children spent more time being sedentary (300.67 ± 44.41 min/day) and less time engaged in light (150.41 ± 36.08 min/day) and moderate-vigorous PA (28.93 ± 15.34 min/day) [52] for 63%, 31% and 6% of the time recorded, respectively.

This inactivity in NZ, and internationally, is concerning relative to effects on cognitive performance. Research suggests that PA [53], physical fitness [54,55], aerobic fitness [56,57] and FMS [58] in children are associated with improved academic performance and cognitive control [53,54,55,56,57,58] and implies that the cognitive development of preschool age children can be stimulated by non-digital games [59]. Furthermore, dose–response effects linking higher volumes of moderate-to-vigorous PA (aerobic PA and cognitively engaging PA) to improved mathematics achievement and higher volumes of moderate-to-vigorous cognitively engaging PA to improved spelling achievement have been reported in school-aged children [60].

Many studies report advances in young children’s FMS, such as object control and locomotor skills, following a PA- or FMS-based intervention [39,40,47,61,62,63]. The implementation of a professional development programme, designed to support ECE teachers to promote young children’s healthy eating and PA, has also significantly improved FMS [64]. Such improvements in FMS have been reported in preschoolers who live with, and those who live without, developmental delays [41,42,65]; these improvements were not seen in a low socioeconomic ECE center with low levels of adherence to the intervention [66]. However, directly comparing the results of such interventions can be difficult because of differences in intervention type, methodology and assessment tools [62]. Therefore, more support and PLD, such as via an FMS intervention, should be provided to ECE educators, especially those in low socioeconomic environments, to improve staff competency and confidence in guiding healthy eating, PA and FMS to contribute to the overall wellbeing and academic performance of preschool children in NZ.

Limitations of this study include the small sample of ECE centers, which is not representative of all ECE in NZ. Furthermore, PA during ECE may not be indicative of total PA across a 24-h period. While we provide a valuable snapshot of the PA and motor skills of children in ECE centers, further studies should include data, such as those which contributed to limitations within this study, including a larger sample size, ethnicity, years of ECE attendance, number of ECE sessions per week, the number of hours of ECE attendance each week, the number of times the accelerometer was worn, urban/peri-urban/rural, different ‘levels’ of quality in the environment, e.g., based on Early Childhood Environment Rating Scale^®^-Revised (ECERS-R), different types of early learning services, e.g., child care, kindergarten, funded playgroups, parent cooperatives, total immersion language, and at-home care/parents, to gain a broader understanding of the PA and motor skills of preschool children in NZ.

Future studies should explore the link between PA, sleep and other factors (e.g., nutrition and screen time) on social-cognitive development in preschool children [67], and explore the effect of an FMS intervention on motor skills in this population.

## 5. Conclusions

Children were primarily inactive, followed by some moderate and little vigorous activity. Gross motor quotient was significantly correlated to locomotor and object control skills, and kCal/hour was significantly related to intensity of activity, but there was no correlation between gross motor quotient and kCal/hour.

## Figures and Tables

**Table 1:** Sample characteristics of participating children.

	Total	Male	Female
Total Participants	84	47 (56%)	37 (44%)
Age (y)	4.02 ± 0.57	3.98 ± 0.62	4.08 ± 0.50
Height (cm)	104.14 ± 5.77	103.20 ± 5.39	105.60 ± 6.26
Weight (kg)	18.74 ± 3.42	18.41 ± 3.45	19.25 ± 3.45
Participated in TGMD-2	81	45 (56%)	36 (44%)
Age (y)	4.00 ± 0.56	3.94 ± 0.60	4.07 ± 0.51
Height (cm)	104.14 ± 5.77	103.20 ± 5.39	105.60 ± 6.26
Weight (kg)	18.74 ± 3.42	18.41 ± 3.45	19.25 ± 3.45
Participated in accelerometry	53	33 (62%)	20 (38%)
Age (y)	4.00 ± 0.56	3.88 ± 0.62	4.21 ± 0.39
Height (cm)	103.68 ± 5.64	102.09 ± 5.28	107.82 ± 4.67
Weight (kg)	18.01 ± 2.29	17.23 ± 1.57	20.04 ± 2.78
Participated in TGMD-2 and accelerometry	50	31 (62%)	19 (38%)
Age (y)	3.97 ± 0.55	3.82 ± 0.58	4.21 ± 0.41
Height (cm)	103.68 ± 5.64	102.09 ± 5.28	107.82 ± 4.67
Weight (kg)	18.01 ± 2.29	17.23 ± 1.57	20.04 ± 2.78

y: years-old; cm: centimeters; kg: kilograms; TGMD-2: Test for Gross Motor Development-2; Categorical data are presented as count (percentage). Mean and standard deviations are presented for normally distributed data. Medians (25th, 75th percentile) are presented for non-normally distributed data.

**Table 2:** Test for Gross Motor Development-2 data.

	Total (*n* = 81)	Male (*n* = 45)	Female (*n* = 36)	*p* Value *
Gross Motor Quotient	104.04 ± 14.53	104.33 ± 14.59	103.67 ± 14.65	0.839
TGMD-2 Locomotor	27.47 ± 10.02	26.73 ± 8.76	28.39 ± 11.45	0.463
Locomotor Standard Score	10.93 ± 3.26	10.76 ± 2.94	11.14 ± 3.66	0.602
Locomotor Percentile	63.00 (37.00, 91.00)	50.00 (37.00, 84.00)	63.00 (37.00, 91.00)	0.509
Locomotor Age Equivalent	4.60 (3.30, 6.00)	4.50 (3.25, 5.50)	4.68 (3.63, 6.25)	0.344
TGMD-2 Object Control Skill	23.46 ± 8.36	25.29 ± 8.74	21.17 ± 7.35	0.026 ^a^
Object Control Standard Score	10.00 (9.00, 12.00)	10.00 (9.00, 13.00)	10.00 (9.00, 12.00)	0.293
Object Control Percentile	50.00 (37.00, 75.00)	50.00 (37.00, 84.00)	50.00 (37.00, 75.00)	0.323
Object Control Age Equivalent	4.25 (3.25, 5.00)	4.75 (3.00, 5.25)	4.25 (3.25, 5.00)	0.588

TGMD-2: Test for Gross Motor Development-2. Mean and standard deviations are presented for normally distributed data. Medians (25th, 75th percentile) are presented for non-normally distributed data. * Independent sample *t*-tests were used for parametric data and Mann–Whitney (Exact sig.) was used for non-parametric data; a level of *p* < 0.05 indicates a significant difference in intakes between male and female. ^a^ medium effect size.

**Table 3:** Accelerometry data.

	Total (*n* = 53)	Male (*n* = 33)	Female (*n* = 20)	*p* Value *
Average kCal per hour	8.65 ± 4.30	8.15 ± 4.10	9.47 ± 4.60	0.468
% of sedentary/light activity	78.31 ± 9.63	77.97 ± 9.57	78.88 ± 9.96	0.984
% of moderate activity	19.55 ± 8.87	19.92 ± 8.90	18.94 ± 9.01	0.827
% of vigorous activity	1.23 (0.83, 2.39)	1.23 (0.78, 2.16)	1.27 (0.90, 2.43)	0.819

Mean and standard deviations are presented for normally distributed data. Medians (25th, 75th percentile) are presented for non-normally distributed data. * Independent sample *t*-tests were used for parametric data and Mann–Whitney (Exact sig.) was used for non-parametric data; a level of *p* < 0.05 indicates a significant difference in intakes between male and female.

**Table 4:** Comparison of Test of Gross Motor Development, 2nd Edition (TGMD-2) scores ^a^.

						Descriptive Rating of Gross Motor Quotient, *n* (%)						
Reference	Group ^b^	Age, Years	Locomotor Standard Scores	Object Control Skills Standard Scores	Gross Motor Quotient	Very Superior (>130)	Superior (121–130)	Above Average (111–120)	Average (90–110)	Below Average (80–89)	Poor (70–79)	Very Poor (<70)
Present study	Total (*n* = 81)	3–4-year-old preschool children, New Zealand	10.93 ± 3.26	10.00 (9.00, 12.00)	104.04 ± 14.53	1 (1.2)	14 (17.3)	11 (13.6)	42 (51.9)	9 (11.1)	4 (4.9)	0 (0)
	Male (*n* = 45)		10.76 ± 2.94	10.00 (9.00, 13.00)	104.33 ± 14.59	1 (2.2)	9 (20.0)	7 (15.6)	20 (44.4)	7 (15.6)	1 (2.2)	0 (0)
	Female (*n* = 36)		11.14 ± 3.66	10.00 (9.00, 12.00)	103.67 ± 14.65	0 (0)	5 (13.9)	4 (11.1)	22 (61.1)	2 (5.6)	3 (8.3)	0 (0)
Aye, Oo [48]	Total (*n* = 472)	5-year-old, kindergarten children, Myanmar	12.8 ± 3.61	10.1 ± 2.81	108.3 ± 16.0	32 (6. 8)	83 (17.6)	95 (20.1)	218 (46.2)	29 (6.1)	12 (2.5)	3 (0.6)
	Male (*n* = 237)		12.9 ± 3.74	10.0 ± 2.65	108.3 ± 16.3	NA	NA	NA	NA	NA	NA	NA
	Female (*n* = 235)		12.6 ± 3.48	10.2 ± 2.95	108.4 ± 15.8	NA	NA	NA	NA	NA	NA	NA
Goodway, Rudisill [50]	Male (*n* = 30)	4-year-old, preschool children, African American	10.43 ± 2.5	NA	NA	NA	NA	NA	NA	NA	NA	NA
	Female (*n* = 29)		11.38 ± 2.69	NA	NA	NA	NA	NA	NA	NA	NA	NA
Kordi, Nourian [47]	Baseline (*n* = 147)	3–6-year-old, preschool children, Iran	9 ± 4	9 ± 3	93.3 ± 18.9	3 (2)	14 (9.5)	8 (5.4)	61 (41.5)	22 (15)	27 (18.4)	12 (8.2)
	Male (*n* = 75)		NA	NA	91.2 ± 17.3	NA	NA	NA	NA	NA	NA	NA
	Female (*n* = 72)		NA	NA	95.5 ± 20.3	NA	NA	NA	NA	NA	NA	NA
Tomaz, Bernstein [49]	Total (*n* = 259)	3–6-year-old, preschool children, South African	11.3 ± 2.5	10.6 ± 2.3	105.5 ± 12.0	4 (1.5)	20 (7.7)	61 (23.6)	156 (60.2)	13 (5.0)	3 (1.2)	2 (0.8)
	Male (*n* = 130)		11.4 ± 2.3	10.4 ± 1.9	105.1 ± 10.2	1 (0.8)	7 (5.4)	31 (23.9)	85 (65.4)	4 (3.1)	2 (1.5)	0 (0)
	Female (*n* = 129)		11.3 ± 2.7	10.8 ± 2.5	105.8 ± 13.6	3 (2.3)	13 (10.1)	30 (23.3)	71 (55.0)	9 (7.0)	1 (0.8)	2 (1.6)
Apache [51]	Baseline 1 (*n* = 28)	3–6-year-old, preschool children with disabilities, Las Vegas	10.2 ± 3.3	8.6 ± 1.7	NA	NA	NA	NA	NA	NA	NA	NA
	Baseline 2 (*n* = 28)		10.9 ± 3.6	9.7 ± 2.8	NA	NA	NA	NA	NA	NA	NA	NA

^a^ Descriptive studies and baseline data. ^b^ Intervention and control groups have been renamed as Baseline 1 and Baseline 2. Scores expressed as mean ± standard deviation or median (25th, 75th percentile). Categorical data are presented as count (percentage). NA: Not applicable.

## Data Availability

All relevant data are presented in this manuscript.
